# Efficiency of Inhaled Cannabidiol in Cannabis Use Disorder: The Pilot Study Cannavap

**DOI:** 10.3389/fpsyt.2022.899221

**Published:** 2022-05-24

**Authors:** Grégoire Cleirec, Esther Desmier, Cristina Lacatus, Simon Lesgourgues, Anais Braun, Claire Peloso, Chanaëlle Obadia

**Affiliations:** ^1^Addiction Support and Prevention Center 110 Les Halles, Groupe SOS Solidarités, Paris, France; ^2^Addiction Department of Hôpital suburbain du Bouscat, Le Bouscat, France; ^3^Addiction Department of René Muret Hospital, Assistance Publique des Hopitaux de Paris, Sevran, France

**Keywords:** cannabis use disorder, vaping, withdrawal, cannabidiol (CBD), harm reduction

## Abstract

**Introduction:**

Cannabidiol (CBD), the second most prevalent cannabinoid found in cannabis, is considered to be safe for use. Studies suggest that CBD may be of benefit in treating cannabis use disorder (CUD). In clinical practice, CBD is already being used by patients who are trying to reduce or stop their cannabis consumption. The aim of this study was to assess the potential of CBD inhaled using a vaping device in CUD.

**Methods:**

This was an exploratory, observational, non-randomized, open-label study conducted at an Addiction Support and Prevention Center in Paris. The primary endpoint was a reduction of at least 50% in the reported number of joints consumed daily at 12 weeks. The participants were given an electronic cigarette along with liquid containing CBD. Nicotine at 6 mg/ml could be added in case of co-consumption of tobacco. They were assessed once a week and the CBD liquid dose was adjusted based on withdrawal signs and cravings (33.3, 66.6 or 100 mg/mL).

**Results:**

Between November 2020 and May 2021, 20 patients were included and 9 (45%) completed the follow-up. All of the participants used tobacco, and were provided a liquid with nicotine. At 12 weeks, 6 patients (30%) had reduced their daily cannabis consumption by at least 50%. The mean number of joints per day was 3, compared to 6.7 at baseline. The mean amount of CBD inhaled per day was 215.8 mg. No symptomatic treatment for cannabis withdrawal was prescribed. Mild adverse effects attributable to CBD and not requiring the prescription of any medicines were reported in a few patients.

**Conclusion:**

This research provides evidence in favor of the use of CBD in CUD. It also highlights the benefits of inhalation as the route of CBD administration in patients who use cannabis: inhalation can allow users to self-titrate CBD based on their withdrawal symptoms and cravings. This study illustrates the interest of proposing an addictological intervention targeting at the same time tobacco and cannabis dependence in users who are co-consumers. A double-blind, randomized, placebo-controlled clinical trial is needed to assess the efficacy of inhaled CBD in CUD.

Study registration number (IDRCB) issued by the ANSM (*Agence nationale de sécurité du médicament et des produits de santé*—French National Agency for Medicines and Health Products Safety): 2018-A03256-49. This study received IEC approval from the CPP Sud-Ouest et Outre-Mer 1 (South-West and Overseas 1 IEC) on 15/06/2020 (CPP 1-19-041/ID 3012).

## Introduction

Cannabis is the most produced and consumed illicit substance in the world. In 2019, the UN estimated that 4% of people aged 15–64 had consumed cannabis and that there were 200 million cannabis users worldwide—an increase of 18% since 2010 (1). In France, 42% of adults have tried cannabis and 11% of people aged 18–64 years are regular users (2). Cannabis smoking leads to increased cough, phlegm, susceptibility to upper respiratory infections, acute psychiatric symptoms such as anxiety and panic and to cannabinoid hyperemesis syndrome in a subset of genetically susceptible people (3, 4). It also increases the risk of cancer and cardiovascular disease when consumed by combustion in association with tobacco. The vast majority of French cannabis users co-consume cannabis and tobacco in the form of joints (5). Most cannabis users therefore have a dual dependency to cannabis and tobacco.

There is currently no specific regulatory-approved treatment for cannabis use disorder (CUD) other than symptomatic treatments for withdrawal symptoms (such as anxiolytics and hypnotics) (6). When chronic users stop using cannabis or reduce their consumption, this can lead to a withdrawal syndrome defined in the DSM-5 as the appearance of three or more of the following symptoms: irritability, anger or aggression; sleep difficulty; decreased appetite or weight loss; restlessness; depressed mood; other physical symptoms such as abdominal pain, tremors, sweating, fever, chills and headaches (7). These symptoms most commonly occur within 24–48 h of withdrawal and can last for up to 4 weeks after cessation of use (6). The Cannabis Withdrawal Scale (CWS) has been validated in English and comprises two subscores ranging from 0 to 190 representing the intensity of withdrawal symptoms and the impact of these symptoms on daily life (8). There are currently no validated scales in French designed to assess withdrawal symptoms or cannabis craving intensity. In clinical practice, visual analog scales (VAS) ranging from 0 to 10 are sometimes used.

Cannabidiol (CBD) is the second most prevalent cannabinoid in cannabis, after Δ-9-tetrahydrocannabinol (THC) (9). Unlike THC, CBD has no hedonic effects (10). Based on the current understanding of CBD, it appears to be a modulator of the endocannabinoid system [a weak antagonist of CB1; (11)], a serotonin receptor agonist *via* the 5-HT1a receptors (12–14) and an allosteric modulator of the μ and δ opioid receptors (15). It may also have an impact on the glutamatergic system (12). Its therapeutic properties are still being studied. It is an anticonvulsant used in particular for certain resistant forms of childhood epilepsy [Dravet syndrome and Lennox Gastaut syndrome; (16, 17)]. Certain studies have identified possible neuroprotective, analgesic, anti-inflammatory, anti-emetic and even anti-cancer effects (18, 19).

The bioavailability of orally administered CBD is 6%, compared to 31% for inhaled CBD (18, 20–22). Peak plasma concentrations are reached 3–10 min after inhalation and are higher than those obtained after ingestion. This route of administration has the advantage of limiting the first pass effect. After inhalation, the elimination half-life is long-−31 h on average (+/−4 h)—and can vary depending on different parameters, such as differences in metabolism, distribution, accumulation in adipose tissue, and biliary and renal excretion (20).

To date, CBD appears to carry a very low risk of toxicity (23–25). The main reported side effects at high doses in studies evaluating CBD in epilepsy were diarrhea, sedation, nausea, headache and changes to appetite. Abnormal liver function tests and pneumonia have also been reported in certain epilepsy studies, but may have been caused by co-administration with anti-epileptic drugs. In a recent metaanalysis, after excluding studies in childhood epilepsy, the only adverse outcome associated with CBD treatment was diarrhea (26). A phase I study involving CBD administration in healthy subjects did not identify any concerning short-term physical or mental effects for CBD doses of up to 6,000 mg daily (27). Furthermore, there is no reported evidence of addictive potential in animal models or humans, and there are no reported cases of CBD misuse.

Numerous psychiatric studies have been conducted into the effects of CBD. CBD appears to have an anxiolytic effect (reduced anxiety with single oral doses of 300–600 mg) (28–31) and an anti-psychotic effect (oral doses of 150–1,500 mg per day) (9, 32). There is scientific justification for a possible anti-depressant effect (9, 12).

From a neurobiological perspective, CBD acts on the endocannabinoid systems involved in the reward pathway *via* the CB1 receptors (11, 33), which suggests that cannabinoids may have therapeutic potential in substance use disorders, regardless of the substance. A potential therapeutic effect of CBD in substance use disorders (opiates, alcohol, smoking, amphetamines, cannabis etc.) can be considered on the basis of preclinical (34, 35) and clinical studies: a reduction in cravings and anxiety was found in former opiate users (36), while the daily number of cigarettes smoked went down when CBD was vaped at a dose of 400 μg (37). However, despite encouraging preclinical and observational data (38), two randomized, placebo-controlled trials did not find any benefits of CBD in cocaine use disorder (39, 40). Several clinical trials have suggested that nabiximols (a sublingual spray containing THC/CBD extracts in a 1:1 ratio) may reduce the intensity of cannabis withdrawal signs (41, 42) and reduce cannabis use both during treatment (43) and for an extended period after treatment cessation (44). Given that studies of dronabinol (THC only) demonstrated its efficacy in reducing withdrawal symptoms but found no effect on abstinence or reduced consumption compared to placebo (45), the reduction in consumption observed with nabiximols may be attributable to the CBD it contains.

To date, only three studies have been published assessing the use of CBD in cannabis use disorder. Two case reports suggest a reduction in cannabis withdrawal symptoms, one with daily oral administration of 400–600 mg CBD tablets (53) and the other with 18–24 mg oral CBD in oil form (46). A phase IIa, randomized, double-blind, placebo-controlled clinical trial was conducted to identify the most effective oral dose of CBD in terms of increased number of days of abstinence from cannabis and lower urinary THC metabolite levels (THC-COOH:creatinine ratio) (47). CBD doses of 400 and 800 mg appeared to be more effective than CBD 200 mg or placebo at reducing cannabis consumption. In addition, CBD appeared to reduce the number of cigarettes smoked and signs of cannabis withdrawal. No differences were found between CBD and placebo in terms of side effects.

The use of electronic cigarettes has been shown to be effective in reducing cigarette consumption and in smoking withdrawal, with a better risk-benefit ratio for vaping (48, 49). In France, the production, sale and use of e-liquids containing CBD is currently legal provided the liquid contains <0.3% THC and the CBD comes from the fiber or seeds of hemp varieties authorized for industrial and commercial use (50). CBD e-liquids are treated as standard consumer goods. In clinical practice, CBD is already being used by patients who are trying to self medicate to reduce their cannabis consumption or stop using cannabis entirely (51).

The use of CBD by inhalation rather than taken orally is particularly interesting given its better pharmacokinetic properties (faster peak plasma levels and better bioavailability). Moreover, the advantage of the electronic cigarette is that it could allow users to self-titrate CBD in the same way that it is used to self-titrate nicotine (52), which would enable each user to adjust their consumption based on their needs and the effects experienced. In a context of limited scientific data on the dosage of CBD that could be effective in CUD, the use of a device allowing self-titration permits to explore the therapeutic potential of this product without being limited by the constraint of fixed doses imposed by the galenic of the tablet. Finally, vape allows for clinical addictological work on the behavioral component of smoked cannabis consumption, which is not possible with CBD tablets or oil.

In the absence of any standard treatment for cannabis withdrawal and given the possibility that CBD may help reduce cannabis consumption in users, it seems pertinent to consider the role CBD vaping might play in the reduction and cessation of cannabis use. No studies have assessed this to date. The aim of this study was therefore to conduct a pilot study to assess the benefits of CBD inhaled *via* an electronic cigarette in reducing or stopping cannabis use.

## Materials and Methods

We conducted an interventional, single-center, non-randomized, uncontrolled, open-label study. It took place at a community addiction facility that offers free, anonymous support on an outpatient basis: the CSAPA (*Center de Soins d'Accompagnement et de Prévention des Addictions*–Addiction Support and Prevention Center) located at 110 les Halles, Paris.

### Endpoints

The primary endpoint was a reduction of at least 50% in reported cannabis consumption, measured in terms of number of joints per day after 12 weeks, compared to reported consumption at baseline.

The secondary endpoints were: daily amount of inhaled CBD, total amount spent on cannabis daily (in euro), cannabis cravings visual analog scale (VAS) score from 0 to 10, withdrawal symptom intensity VAS score, the two CWS subscores, number of cigarettes smoked per day, exhaled carbon monoxide level measured using an electronic device, occurrence of adverse effects and prescription of symptomatic treatments for signs of cannabis withdrawal.

### Participants

To participate in the study, the patients had to be adults with cannabis use disorder (based on the DSM-5 substance abuse disorder criteria), have health insurance/social security coverage, have contacted the CSAPA at 110 les Halles, Paris in the hope of reducing or stopping their cannabis use, and test positive for THC in a urine toxicology test at the first medical consultation.

The exclusion criteria were the presence of any acute psychopathology, legal guardianship, a use disorder for any substance other than cannabis (except cigarettes) and pregnancy and/or breastfeeding. Participation in the study was free, anonymous and at no financial cost to the participant.

The number of subjects to include was arbitrarily set at 20, given the modest human and financial resources at our disposal for the study.

### Follow Up Process

Patients interested in participating in the study met with one of the study doctors, who provided them with clear, comprehensive information. The inclusion and exclusion criteria were also checked during this meeting. The patients were given a written information sheet. After a reflection period of at least 48 h, they came in for a baseline visit, during which they signed a consent form.

**Figure 1:**
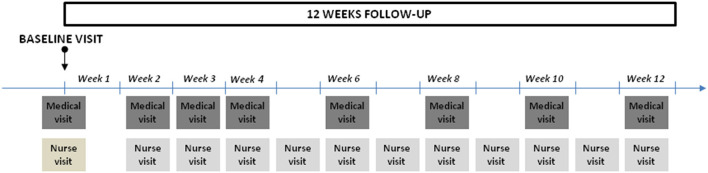
Timeline of the follow-up.

The follow-up lasted 12 weeks in total (Figure 1). The participants attended eight medical visits, the first four at 1 week intervals and the rest every 2 weeks. The following were assessed at each visit: daily joint consumption, total amount spent on cannabis in euros, signs of cannabis withdrawal (irritability, anger, sleep difficulty, decreased appetite or weight loss, restlessness, depressed mood; abdominal pain, tremors, sweating, fever, chills and/or headaches), side effects attributable to CBD, number of cigarettes smoked per day and exhaled carbon monoxide. Symptomatic treatment for signs of cannabis withdrawal and nicotine replacement therapy could be prescribed for the duration of the study.

After the first medical visit, the patients had a consultation with a nurse to teach them how to use the electronic cigarette. At the end of this consultation, they were given a 30-mL bottle of liquid containing 33.3 mg/mL CBD, with or without nicotine at 6 mg/mL, with a flavor of their choice (tobacco, red berries or cannabis). Obtaining a liquid with nicotine was recommended in case of tobacco consumption to limit the risk of increased cigarette consumption or signs of tobacco withdrawal, but left to the choice of the participants. The liquids were purchased from the compagny Leaf, which produced them specifically for the study. The starting CBD dose of 33.3 mg/mL was selected based on clinical trials of CBD in anxiety, which identified an anxiolytic effect of CBD at oral doses of 300 mg and over. The administered CBD dose was reassessed every seven days (at the pharmacodynamic steady state, i.e., after five CBD half-lives). At this point, one of the study nurses also interviewed the patients to assess daily CBD consumption, the number of joints smoked per day, cannabis cravings VAS score, withdrawal signs VAS score and CWS score. The use of the vaping device was also assessed regularly, new coils could be provided and patients were reminded how to use the electronic cigarette, where necessary. The CBD liquid dose was adjusted depending on these data: it could be increased to the higher dose (if the cannabis cravings VAS score was ≥ 5/10 and/or the withdrawal signs VAS score was ≥ 5/10), reduced to the lower dose (cannabis cravings ≤ 2/10 and withdrawal signs ≤ 2/10) or continued at the same dose in the other possible scenarios. We settled for these cut-off values because they seemed to us clinically relevant. Three different concentrations of CBD were available, with nicotine or without nicotine at a single dose of 6 mg/mL: 33.3 mg CBD per mL, 66.6 mg CBD per mL, and 100 mg CBD per mL. The participants could visit the CSAPA between the follow-up nurse visits if they had any problems using the electronic cigarette of if they needed to collect more CBD liquid at the same concentration.

### Statistical Analysis

Analyses were performed using an observed method. Because of the small cohorts, we report no inferential statistical analyses; outcomes are summarized by descriptive statistics. All analyses were performed using R Studio 4.0.0® or higher.

For the primary endpoint, descriptive statistics were performed for the last visit across all doses of CBD. Those lost to follow up were deemed not to have met the primary endpoint

For secondary endpoints, descriptive analysis was conducted, using a Chi-square or Fisher test, to identify factors impacting on the primary endpoint. The missing data were not imputed for secondary endpoints.

## Results

**Figure 2:**
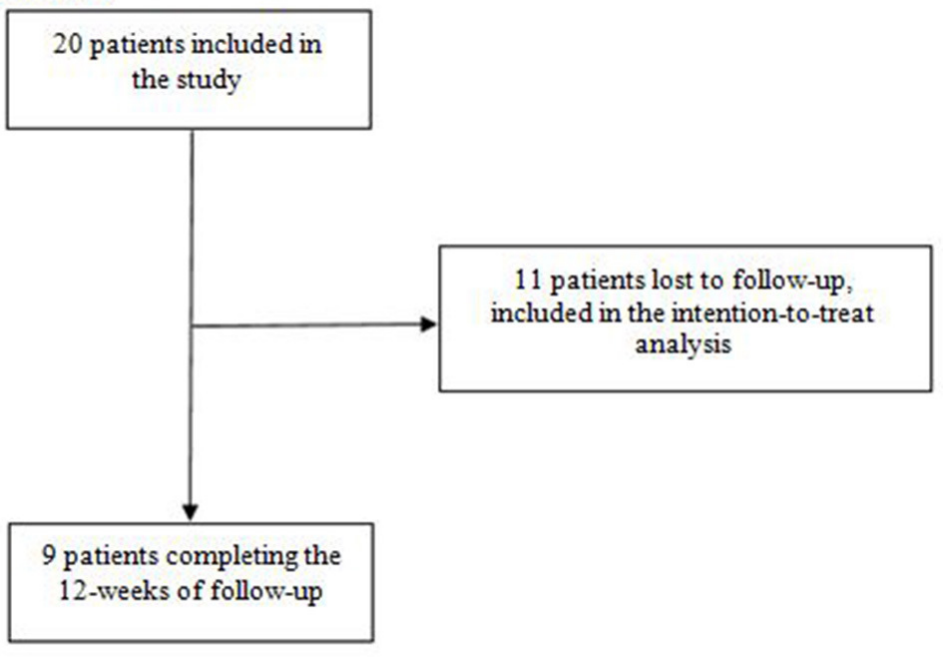
Flow chart.

The study took place from 10/09/2020 to 05/12/2021. Twenty patients were included, and nine participants (45%) completed the full 12 weeks of follow-up (Figure 2).

### Sample Characteristics

The patients were mostly men (90%), young (mean age 36 years, youngest 21 years and oldest 61 years), single (80%) and in employment (65%) (Table 1). The majority of the patients had a history of psychiatric illness (65%): either a mood or anxiety disorder. Fifty percent of the patients had a history of a use disorder for a substance other than cannabis or cigarettes. Eighty percent of the patients were smokers, most of them had already tried a vaping device (75%) and half had previously tried CBD. All the patients consumed cannabis solely in the form of joints containing both cannabis and tobacco, with the exception of one patient who smoked it using a water pipe (bong). The mean number of joints consumed per day was 6.7 (minimum 1.5, maximum 20). Two thirds of the patients were aiming for abstinence, while the other third wanted to control their use.

**Table 1:** Participant characteristics.

**Characteristics**	***N* = 20^***a***^**
Gender	
Male	18 (90%)
Female	2 (10%)
Age	36 (32,48)
Marital status	
Single	16 (80%)
Partnered	4 (20%)
Employment category	
Farmer	0
Tradesperson/retailer, small business owner	5 (25%)
Highly educated professionals and managers	8 (40%)
Middle managers, teachers and non-managerial health and social care professionals	0
Non-managerial employees	4 (20%)
Manual workers	3 (15%)
Retired - economically inactive	0
In employment	
No	7 (35%)
Yes	13 (65%)
Housing	
Home owner	3 (15%)
In rental accommodation	13 (65%)
In hostel	3 (15%)
Homeless	1 (5%)
Health insurance coverage	
CPAM [national health insurance] and private health insurance	10 (50%)
CPAM and no private health insurance	1 (5%)
CMU [universal health insurance] and private health insurance	1 (5%)
CMU and CSS [additional health insurance for people on a low income]	6 (30%)
AME [state medical aid for undocumented immigrants]	2 (10%)
Psychiatric comorbidity	
None	7 (35%)
Mood disorder	9 (45%)
Anxiety disorder	4 (20%)
Psychosis	0
Cigarette smokers	16 (80%)
Previous use of vaping device	16 (75%)
Previous use of CBD	10 (50%)
Cannabis use characteristics	
Age at first use	16 (12,21)
Age at loss of control	25 (18,30)
Product type/method of consumption	
Resin	6 (30%)
Cannabis buds	1 (5.0%)
Resin and buds	13 (65%)
Method of consumption	
Joint	19 (95%)
Vaporiser	0
Other	1 (5%)
Number of joints per day	6.7 (4.8, 8.5)
Total spent per day (€)	10.75 (0, 23)
Addiction objective	
Abstinence	13 (65%)
Controlled use	7 (35%)

a *n (%); mean (IQR)*.

### Primary Endpoints

At the end of the 12 weeks of follow-up, nine patients were still being followed up. Six patients (30% of the participants) had reduced their cannabis consumptions by at least 50% (Table 2), accounting for 67% of the 9 participants still being followed up at 12 weeks.

**Table 2:** Change in primary endpoint 1.

	**Week 2**	**Week 3**	**Week 4**	**Week 6**	**Week 8**	**Week 10**	**Week 12**
**≥50% reduction in daily joints** ^***a***^	6 (30%)	5 (25%)	8 (40%)	6 (30%)	5 (25%)	4 (20%)	6 (30%)
**≤50% reduction in daily joints** ^***a***^	14 (70%)	15 (75%)	12 (60%)	14 (70%)	15 (75%)	16 (80%)	14 (70%)
**Total number of patients still followed up**	18	15	15	12	11	9	9
**Lost to follow-up**	2	5	5	8	9	11	11

a *n (%) 1*.

### Secondary Endpoints

After 12 weeks of follow-up, the mean number of joints consumed per day was 3 (minimum = 0, maximum = 7), compared to a mean of 6.7 joints per day at baseline for all of the participants, and a mean of 5.44 joints per day at baseline for the 9 participants who ended up completing the follow-up. Three participants had stopped using cannabis entirely. The mean total amount spent on cannabis per day was €4.40, compared to €10.75 at baseline. The participants soon needed to increase the concentration of CBD in their liquid (Table 3). The mean amount of CBD inhaled per day was 215.8 mg at 12 weeks. No symptomatic treatment for cannabis withdrawal was prescribed, since only mild signs of withdrawal were observed during the medical follow-up.

**Table 3:** Change in CBD consumption during follow-up.

	**Week 2**	**Week 3**	**Week 4**	**Week 6**	**Week 8**	**Week 10**	**Week 12**
**33.3 mg/mL liquid** ^***a***^	18 (100)	7 (47)	2 (13)	2 (17)	0	0	0
**66.6 mg/mL liquid** ^***a***^	0	7 (47)	10 (67)	2 (17)	3 (27)	1 (11)	1 (11)
**100 mg/mL liquid** ^***a***^	0	1 (6.7)	3 (20)	8 (67)	8 (73)	8 (89)	8 (89)
**Total number of participants**	18	15	15	12	11	9	9
**Lost to follow-up**	2	5	5	8	9	11	11
**CBD consumed by participants (mg/day)** ^***b***^	56 (48)	123 (130)	149 (129)	190 (197)	190 (197)	235 (190)	216 (125)

a *n (%)*;

b *mean (standard deviation)*.

No participants returned negative THC urine tests during the study.

With regards to smoking, all the patients chose fluid containing nicotine. Four (20%) received nicotine replacement therapy. The mean number of cigarettes smoked per day was 2.67 per participant at 12 weeks, compared to 7 at baseline. At 12 weeks, 2 of the patients who were smokers at baseline had stopped smoking cigarettes. The participants' exhaled carbon monoxide levels decreased compared to baseline: 50% of the patients (4) had a CO level below 10 ppm at 12 weeks compared to 26.3% at baseline (Table 4).

**Table 4:** Change in consumption between baseline and 12 weeks.

**Characteristics**	**Baseline** **(*N* = 20*^***a***^*)**	**12 weeks** **(*N* = 20*^***a***^*)**
Number of joints per day	6.70 (3.42)	3 (3)
Lost to follow-up		11
Total amount spent on cannabis per day (€)	10.8 (5.8)	4.2 (4.8)
Lost to follow-up		11
Daily concentration of inhaled CBD (mg/day)	56 (48)	216 (125)
Lost to follow-up		11
Carbon monoxide level		
0 to 4 ppm	4 (21%)	2 (25%)
5 to 9 ppm	1 (5,3%)	2 (25%)
10 to 14 ppm	5 (26%)	3 (38%)
15 to 24 ppm	5 (26%)	0 (0%)
> 24 ppm	4 (21%)	1 (12%)
Lost to follow-up		12
Number of cigarettes per day	7 (6)	2.67 (2.50)
Lost to follow-up		11

a *Mean (SD); n (%)*.

Mild adverse effects attributable to CBD and not requiring the prescription of any medicines were reported in 12 patients (60% of participants): irritation of the upper airways with or without cough in seven patients (35%), four of whom were lost to follow-up and three of whom reported that this symptom disappeared quickly during the follow-up period; temporary fatigue in 6 patients (30%); and self-limiting diarrhea in one patient (5%). At each visit throughout the follow-up period, the majority of patients presented with no adverse effects.

### Subgroup Analysis

At 4 weeks, the six participants who reduced their daily joint consumption by at least 50% were consuming more CBD than the other participants: 221 mg/day compared to 66 mg/day (with mean consumption of 149 mg/day inhaled CBD for all the participants). There were no differences between these two groups at 8 weeks (190 mg/day on average) or at 12 weeks (215 mg/day on average).

At week 12, the patients who had reduced their cannabis consumption by at least 50% had a lower mean CWS withdrawal intensity score (19.83) than the group that had not reduced their consumption by 50% (62.33), with a mean overall score of 34 for all the participants who were still being followed up. They also had a lower mean CWS impact on daily life score (3.5) compared to the other group (8), with an overall mean of 5 for all the participants. The mean cannabis withdrawal symptoms VAS score was also lower in the subgroup that reached the primary endpoint: 0.83 compared to 6.33 in the other group, with an overall mean of 2.67.

## Discussion

This pilot study is the second clinical study to assess the benefits of CBD in cannabis use disorder (CUD). It is the first clinical study to explore the inhalation of CBD *via* a vaping device in a substance use disorder. At the end of the 12-week follow-up period, 6 users (30% of the participants) had managed to reduce their cannabis consumption by at least 50%. All participants chose the option of adding nicotine in the liquids. Although this is a pilot study involving a small number of participants, this research provides evidence in favor of the use of CBD in CUD. It also shows that people with CUD can use an electronic cigarette as a tool to reduce their cannabis consumption, and that it is possible to support them with this at an outpatient addiction center.

One of the aims of our study was to assess the amount of inhaled CBD required to reduce consumption. Since the bioavailability of inhaled CBD is 3–4 times higher than that of oral CBD, we used a liquid with a concentration of CBD that would enable users to vape around 100 mg CBD per day (i.e., 3 mL of liquid dosed at 33.3 mg/mL), which would seemingly correspond to an anxiolytic dose (26, 27, 29). The mean daily inhaled CBD consumption per patient was 215.8 mg, equivalent to 3.24 mL of liquid dosed at 66.6 mg/mL. After 4 weeks of follow-up, the group of participants who had reduced their cannabis consumption by at least 50% had a higher mean CBD consumption than the other participants (221 mg/day). This difference then disappeared, with the mean for both subgroups converging around 200 mg/day. It may therefore be worth advising someone who wants to use CBD to reduce their cannabis consumption to use a liquid with a high concentration of CBD. Very few of our participants used the 33.3 mg/mL liquid after the start of the follow-up. If a larger scale study were to be conducted, we would suggest using liquids with a minimum concentration of 60 mg/mL. In April 2022 in France, a vial of 10 ml of liquid with a CBD concentration of 60 mg/ml cost between 18 and 30€, which corresponds to a price of 6 to 10€ for one person to inhale 200 mg of CBD per day. In practice, there is significant variability in the amount of CBD absorbed by each patient, depending on how they vape (amperage of the electronic cigarette coil, selected wattage, method of inhaling etc.). This limits the relevance of the “amount of CBD consumed per day” variable calculated simply as a function of the amount of liquid vaped per day and the concentration of CBD in the liquid. However, the interest of the inhalation by electronic cigarette is that each user can control the quantity of CBD absorbed according to the quantity of liquid consumed and its way of vaping by auto-titration (52).

The fact that no treatments were prescribed for cannabis withdrawal symptoms suggests that CBD is effective against these symptoms. The protocol did not specify when medicines for these symptoms should or should not be prescribed; this was left to the discretion of the two study doctors. It is also possible that the patients had few signs of withdrawal due to the gradual nature of the reduction in their consumption (or due to a lack of reduction, in some cases).

The patients who reached the primary endpoint at 12 weeks had less intense cannabis withdrawal symptoms, according to the VAS and CWS, and these symptoms had less of an impact on their daily life, as measured by the CWS. Our initial hypothesis is that these were “responder” patients, in whom CBD satisfactorily calmed withdrawal signs, either for reasons relating to interpersonal variation in response to this substance, or for reasons relating to interpersonal variability in withdrawal symptoms. It would appear likely, for example, that CBD would better soothe a patient whose main cannabis withdrawal symptom was anxiety than a patient whose main symptom was insomnia. A second hypothesis is that some of these participants had reduced their consumption at the start of the follow-up and were therefore distanced from any cannabis withdrawal symptoms that they may have experienced (since these symptoms last <1 month from withdrawal). Patients whose consumption fluctuated between reductions and increases would have cannabis withdrawal symptoms for longer than patients who managed to quickly reduce or stop their use.

The most common CBD adverse effect—irritation of the airways, reported by one third of the participants—was usually temporary. It is likely that this irritation was partly related to difficulty using the electronic cigarette, and that regular reminders on how to use the device resulted in the symptom going away. This symptom could also be imputed to the nicotine present in the liquid. The second most frequently reported adverse effect was temporary fatigue, in 30% of patients. It is difficult to identify whether this effect was fully attributable to the CBD or partially linked to the cannabis withdrawal itself. However, all the adverse effects were mild, which provides additional evidence suggestive that short-term CBD vaping is safe. More widely, there is scientific controversy regarding the risk of long-term electronic cigarette use (53–55). The inhalation of CBD *via* an electronic cigarette should therefore be considered a short-term or transitional option. Furthermore, since the possible mechanisms of action of CBD in CUD are rooted in a reduction of withdrawal symptoms and cravings, the administration of CBD over long periods would not appear to be necessary. Contrary to the perceptions of some CBD users, we do not believe that CBD is the equivalent of opiate replacement therapy for cannabis.

Our study has several biases. It is an exploratory, non-randomized, uncontrolled, open-label study with a small number of participants. It therefore provides some new information, but cannot offer conclusive evidence on the efficacy of inhaled CBD in CUD. The aim of this study was to assess its feasibility in order that a second, larger scale, multi-center, randomized, placebo-controlled study could be conducted if the conclusions suggested that CBD may be effective. Some of the results will enable us to refine aspects of the protocol, for which there were no available scientific data to draw on at the time of its design, such as the daily CBD dose to administer in order to hopefully achieve clinical efficacy. A study has since provided data on this point for oral CBD (47).

The study has a selection bias, evident in the proportion of participants who had already tried a vaping device (75%), although only a minority were active vape users at the start of the study. The proportion of participants who had previously tried CBD was also high (50%). This may have improved treatment retention in patients who were already familiar with vaping and CBD.

This study presents a confounding bias related to the French cultural particularity of co-consuming cannabis with tobacco. We therefore chose to offer the option of adding nicotine in the vaping liquid to adapt to the practices of our target population. We considered that it was unethical not to propose nicotine to accompany the tobacco withdrawal which would inevitably take place with the reduction of consumption in joints, and to prevent an increase in the cigarette consumption of the participants. Indeed, in our population, only one participant didn't use cannabis with tobacco in joints (he smoked it in a pipe), and he was also a cigarette smoker. We observed in fact two cases of smoking cessation, a reduction in the mean number of cigarettes smoked per day by the participants and a decrease in exhaled carbon monoxide levels during the follow-up period. These effects may have been related to the presence of nicotine in the liquids used and the addiction support on offer, plus an addictolytic effect of CBD in smoking, as discussed in the scientific literature (37).

There could be another confounding bias due to the important proportion of participants with a history of psychiatric illness (65% of our population). It is possible that CBD may reduce some psychiatric symptoms, especially anxiety, and that it is through this indirect mechanism that participants with psychiatric conditions reduce their cannabis consumption This confounding bias was mitigated by the need to be psychiatrically stable to be included in the study. To verify this hypothesis in a larger study, it would be interesting to check if there is a greater proportion of psychiatric illnesses in the participants who respond to CBD.

Clinical studies in CUD share a number of methodological limitations that we also experienced, in particular the difficulty of measuring the amount of cannabis consumed by participants. We do not believe that the proposal to establish a “standard THC unit” (56) is currently applicable to the French context, in which cannabis is obtained illegally, with a high level of variability in product composition and individual consumption practices. While the content of a joint consumed by a given patient is not necessarily comparable to that of another, we decided that this was the unit of measurement for cannabis best suited to our study population. In particular, we were not convinced that all the patients would be able to assess their daily cannabis consumption in grams, and this would still be an unreliable unit of measurement given the variability in the type of cannabis purchased (resin or bud) and in the cannabis content of resin. Another major challenge is how to determine clinically relevant endpoints other than abstinence. A scientific consensus appears to be emerging around the value of research assessing a reduction in consumption rather than abstinence, while acknowledging the difficulty in finding useful endpoints (45, 57, 58): reduction in quantity consumed, reduction in frequency of use, improvement in quality of life, improvement in dependence severity scales etc. However, there is no consensus as to what constitutes a clinically significant reduction in cannabis consumption. In clinical trials assessing the efficacy of vaping or other measures to reduce smoking, the primary endpoint is most commonly a reduction of at least 50% in the number of cigarettes smoked per day (59, 60). We therefore settled on a primary endpoint of a reduction of at least 50% in the amount of cannabis consumed in terms of number of joints per day. The fact that this primary endpoint is based on self-reporting introduces a potential risk of social-desirability bias, although clinical trials in substance use disorders have shown generally high levels of consistency between data reported by participants and objective toxicological data (57, 61). It is interesting to note that despite the undeniable value of aiming for harm reduction, the majority of participants (65%) said at the beginning of the study that they wanted to achieve abstinence from cannabis.

The use of qualitative urine toxicology tests proved to be of limited value during the study, since three patients stopped using cannabis completely, and this occurred at the end of the follow-up, which meant there was insufficient time to check that the urine tests were negative. However, the urine tests would have allowed us to discuss any undeclared consumption of substances other than cannabis with the patient, although this situation did not arise during the follow-up.

Treatment retention was 45% at 12 weeks. This figure is comparable to the retention rates in other clinical studies of patients with CUD (42, 43, 62). This is particularly noteworthy given that the research took place during the COVID-19 pandemic: the French health authorities imposed two strict lockdowns on the French population during the study period, which made it much more complicated to offer outpatient support to the participants.

This research highlights the benefits of CBD in CUD and need to continue evaluating this substance. It also illustrates the benefits of inhalation as the route of CBD administration in patients who already consume cannabis: inhalation can allow users to self-titrate CBD based on their withdrawal symptoms and cravings. A double-blind, randomized, multi-center, placebo-controlled clinical trial is still needed to assess the efficacy of inhaled CBD in CUD.

## Data Availability Statement

The raw data supporting the conclusions of this article will be made available by the authors, without undue reservation.

## Ethics Statement

The studies involving human participants were reviewed and approved by CPP Sud-Ouest et Outre-Mer 1 (South-West and Overseas 1 IEC). The patients/participants provided their written informed consent to participate in this study.

## Author Contributions

GC designed the study and wrote the first draft of the manuscript. GC and CO organized the database. ED, CL, SL, and AB collected the data. CO performed the statistical analysis. All authors contributed to manuscript revision, read, and approved the submitted version.

## Funding

This study was carried out with SOS Solidarités' own funds.

## Conflict of Interest

The authors declare that the research was conducted in the absence of any commercial or financial relationships that could be construed as a potential conflict of interest.

## Publisher's Note

All claims expressed in this article are solely those of the authors and do not necessarily represent those of their affiliated organizations, or those of the publisher, the editors and the reviewers. Any product that may be evaluated in this article, or claim that may be made by its manufacturer, is not guaranteed or endorsed by the publisher.
